# Aggressive Natural Killer Cell Leukemia: A Brief Overview of Its Genomic Landscape, Histological Features, and Current Management

**DOI:** 10.7759/cureus.22537

**Published:** 2022-02-23

**Authors:** Vikram Sumbly, Mallorie Vest, Ian Landry

**Affiliations:** 1 Internal Medicine, Icahn School of Medicine at Mount Sinai, New York City Health and Hospitals/Queens, Jamaica, USA; 2 Medicine, Icahn School of Medicine at Mount Sinai, New York City Health and Hospitals/Queens, Jamaica, USA

**Keywords:** natural killer cells, epstein barr virus, leukemia, ankl, aggressive natural killer cell leukemias(ankl)

## Abstract

Aggressive natural killer-cell leukemia (ANKL) is a rare hematological malignancy characterized by the abnormal proliferation of natural killer (NK) cells. There are currently no therapies approved by the US Food and Drug Administration (FDA) for the treatment of ANKL, but advancements in genomics are assisting in the unraveling of this rare malignancy. We selected 37 articles that contained information on genomics, immunohistochemistry, and/or current clinical trials relating to the treatment and survival of ANKL. Current therapeutic strategies have been subdivided into (1) concurrent chemoradiation, (2) sequential chemoradiation, and (3) sandwich chemoradiation. These methods have been developed to reduce toxicity while still producing a pathologic response. Concurrent chemoradiation with VIDL (etoposide, ifosfamide, dexamethasone, and L-asparaginase) produced an excellent clinical response, while sequential chemoradiation with SMILE (steroid dexamethasone, methotrexate, ifosfamide, L-asparaginase, and etoposide) showed an adequate response, but with severe hematologic toxicity. The efficacy of L-asparaginase in chemotherapeutic regimens and its association with NK-cell apoptosis have led to its inclusion in all standard regimens. Future studies are focusing on the addition of a programmed death-ligand 1 (PD-L1) inhibitor and hematopoietic stem cell transplant (HSCT).

## Introduction and background

Aggressive natural killer-cell leukemia (ANKL) is a rare hematological malignancy characterized by the abnormal proliferation of natural killer (NK) cells. In the 2016 World Health Organization (WHO) classification, ANKL is classified as a type of "Mature T and NK neoplasm" and has an association with the Epstein-Barr Virus (EBV) [1]. Males are twice as likely as females to be diagnosed with ANKL, with an age distribution that favors older populations, except for young adult Asian males [2-5]. The median overall survival (mOS) of ANKL is seven months, with decreasing survival rates from 45% to 31% from one year to five years, respectively [5]. Racial disparities exist in survival as African American patients are more likely to have poorer outcomes [5]. Although there are currently no therapies approved by the US Food and Drug Administration (FDA) for the treatment of ANKL, advancements in the field of genomics have considerably assisted scientists in better understanding this malignancy. Here, we present a brief overview of ANKL with consideration of its genomic landscape and histologic features, with a discussion of the current treatment regimens and the challenges experienced in the treatment of this aggressive, deadly disease.

## Review

Methods

The two authors conducted the informal search on November 1, 2021, via PubMed, Science Direct, Google Scholar, and Clinical Trials search engines. The following keywords were used for the review: "natural killer cell leukemia" and "ANKL" (Table 1).

**Table 1:** Number of articles per search engine for specific terms

Keyword	PubMed	ScienceDirect	Google Scholar
Aggressive natural killer-cell leukemia	609	3,978	1,690
ANKL	63	168	1,800

The selected articles were basic science, clinical research, or translational research papers. Studies were included if they fulfilled any of the following criteria: (1) scientific papers written in English between 2010 and 2021; (2) published in vivo or in vitro studies evaluating the genomics of ANKL; (3) published clinical research which evaluated chemotherapeutic regimens and their impact on progression-free survival. The majority of articles that were removed did not contain information on etiology, treatment, or outcomes. After a screening process that removed duplicate articles, a total of 37 articles were selected for this review as they contained information on the genetic landscape, immunohistochemistry, and/or current clinical trials. Eight of these articles evaluated current chemoradiation therapy and progression-free survival and are summarized in Table 2.

**Table 2:** A review of current treatment regimens in ANKL with corresponding survival PFS: progression-free survival The table is adapted from Kim et al. [14], Journal of Hematology-Oncology 2018.

Study, year	Regimen	Patients (n)	Included drugs	PFS
Concurrent chemoradiation				
Yamaguchi et al. [6]	DeVIC	27	Dexamethasone, etoposide, ifosfamide, carboplatin	57%, 5-year
Michot et al. [7]	ESHAP	13	Etoposide, steroid, Ara-C, cisplatin	72%, 2-year
Tsai et al. [8]	DEP/DVIP	33	Dexamethasone, etoposide, cisplatin/dexamethasone / etoposide, ifosfamide, cisplatin	60%, 5-year
Weekly cisplatin with radiation followed by chemotherapy				
Kim et al. [9]	VIDL	30	Etoposide, ifosfamide, dexamethasone, L-asparaginase	73%, 5-year
Sequential chemoradiation				
Kwong [10]	SMILE	17	Dexamethasone, methotrexate, ifosfamide, L-asparaginase, etoposide	NA
Dong et al. [11]	DICE-L	33	Cisplatin, ifosfamide, etoposide, dexamethasone, L-asparaginase	89%, 5-year
Sandwich chemotherapy				
Wang et al. [12]	GELOX/PGEMOX	27	Gemcitabine, L-asparaginase, oxaliplatin/pegaspargase, gemcitabine, oxaliplatin	86%, 2-year
Li et al. [13]	GELOXD/GEMOXD	167	Gemcitabine, L-asparaginase, oxaliplatin, dexamethasone/pegaspargase, gemcitabine, oxaliplatin, dexamethasone	73%, 5-year

Discussion

Etiology

The exact etiology of ANKL remains poorly understood because it is an extremely rare entity. The Epstein-Barr virus (EBV) seems to play an important role in the pathobiology of this malignancy and is thought to be one of many factors responsible for its aggressive nature [15]. It is postulated that the EBV aids neoplastic NK cells in avoiding activated cytotoxic T-cells via a type I latency pattern of infection [16]. Surprisingly, a few cases of EBV-negative ANKL have also been reported. Unlike EBV-positive ANKL, EBV-negative ANKL tends to occur in middle-aged adults and does not display a racial preference [17,18]. Otherwise, EBV-negative ANKL patients possess similar symptoms and clinical courses as their EBV-positive counterparts [17].

Genomic Landscape

Preliminary results obtained by Song et al. indicated EBV-positive ANKL cells were susceptible to mutations in various immunodeficiency genes such as FANCA p.S1088F, TGFBR2 p.T315M, IRF7 p.K446R, PRF1 p.R4C, MPEG1 p.P316S, and others [19]. EBV-positive ANKL cells were also susceptible to different chromosomal aberrations such as dup1(q22q25), inv(3)(p21p25), del(3)(p13), t(3;11)(q21;q23), i(7)(q10), del(7)(q32), del(14)(q24), and der(15)t(7;15)(q10;q10) [18]. With the help of whole-genome sequencing, ANKL patients (n=29) were found to harbor various mutations in the TP53 (34%), TET2 (28%), CREBBP (21%), and MLL2 (21%) genes, respectively [18]. All included patients had abnormally elevated levels of the extracellular STAT3 stimulator interleukin (IL)-10, while 48% (14/29) of them harbored mutations in the Janus kinase and signal transduction activation of transcription (JAK-STAT) pathway [19]. More specifically, Huang et al. noticed that ANKL patients were prone to STAT3 phosphorylation and increased expression of the transcription factor MYC, which is under the control of JAK and STAT genes [20]. This JAK-STAT-MYC biochemical cascade is thought to be important for the activation of nucleotide synthesis and glycolysis in ANKL cells [20]. Dufva et al. studied the genomes of 14 ANKL patients and identified several mutations in epigenetic modifiers (50%), DDX3X (29%), STAT3 (21%), and RAS-MAPK pathway genes (21%) [21]. Although El Hussein et al. found similar mutations in their ANKL patients, they also detected mutations in TP53 (3/6; 50%), TET 2 (33%), and ASXL1 (33%) [22].

Clinical Course

Most ANKL patients experience a constellation of symptoms. The presence of B-symptoms (e.g., fevers, night sweats, unintentional weight loss, and malaise), lymphadenopathy, and hepatosplenomegaly are signs of circulating disease [23]. The clinical course is often fulminant as patients rapidly develop coagulopathies and hemophagocytic lymphohistiocytosis (HLH), followed by multiple organ failure [23]. The overall prognosis remains grim, with most patients surviving only a few months [24]. 

Histological Features and Immunohistochemistry

Leukemic NK cells resemble or are slightly larger than large granular lymphocytes (LGL) [15]. These neoplastic cells routinely display irregular nuclear contours, nuclear enlargement, and an open chromatin pattern with prominent nucleoli [15,17,25]. The cytoplasm is more likely to be pale or slightly basophilic and contains multiple azurophilic granules of varying prominence [25]. According to El Hussein et al., malignant NK cells can affect the bone marrow subtly or prominently and typically have two patterns of infiltration: sinusoidal or interstitial [16,22].

ANKL cells have an increased expression of CD56 and CD94 [7]. It is theorized that the CD56 surface antigen, a neural cell adhesion molecule, aids in the process of metastasis [16]. The transmembrane-anchored glycoprotein CD94 binds with NKG2A, the inhibitory checkpoint receptor for human leukocyte antigen (HLA)-E, to form functionally distinct heterodimers [25]. Once bound to its ligand (HLA)-E, the CD94-NKG2A complex serves as an interface that allows NK cells to monitor the expression of other HLA molecules [26]. It is hypothesized that irregularities in the expression of the CD94-NKG2A complex can lead to tumor resistance to immune cells [27]. ANKL cells are known to positively stain for the following markers: CD2, CD3ε, CD16, CD56, CCR5, TIA-1, perforin A, and granzyme B [28]. These markers are also found on mature CD56+ NK cells.

Current Treatments and Clinical Trials

Traditionally, ANKL treatment consisted of anthracycline-containing chemotherapy followed by local radiation therapy of at least 45 Gy [29]. This regimen was the standard of care for locally aggressive NK/T-cell lymphomas. However, the utilization of this regimen in ANKL resulted in poor five-year survival rates (30-40%), effectively showing that these aggressive phenotypes did not respond to anthracyclines [30]. This resistance is hypothesized to be secondary to high concentrations of P-glycoprotein, a known resistance factor to anthracycline therapy [29,30]. In 2005, Yamaguchi et al. studied concurrent chemoradiation with DeVIC chemotherapy (carboplatin, etoposide, ifosfamide, and dexamethasone), which found good local control of tumor burden without significant toxicity [29]. However, multidrug resistance remained a significant obstacle, which led to the development of new chemotherapeutic regimens (Table 2).

Treatment has mostly been subdivided into three techniques: (1) concurrent chemoradiation, (2) sequential chemoradiation, and (3) sandwich chemoradiation, in which chemoradiation is followed by additional cycles of chemotherapy. With the modest success of DeVIC, concurrent chemoradiation was further explored with ESHAP (etoposide, steroid, Ara-C, and cisplatin) and DEP, both of which were somewhat unsuccessful secondary to hematologic toxicity [31,32]. Concurrent chemoradiotherapy with VIDL (etoposide, ifosfamide, dexamethasone, and L-asparaginase) showed a pathologic complete response of 87% with progression-free survival at five years of 73% [33].

Sequential chemoradiation began with the creation of SMILE (steroid dexamethasone, methotrexate, ifosfamide, L-asparaginase, and etoposide) [29,30]. Patients treated with SMILE had an excellent 90% response rate but had severe hematologic toxicity, which led to the recommendation of its avoidance in elderly and frail patients. Etoposide had proven efficacy in EBV-related NK lymphoma [29,30]. Methotrexate and ifosfamide were found to be unaffected by P-glycoprotein drug resistance [31]. L-asparaginase was shown to selectively lead to apoptosis in NK cells due to its depletion of tumor essential L-asparagine [31]. The efficacy of L-asparaginase prompted inclusion in several combination regimens, including AspaMetDex (L-asparaginase, methotrexate, and dexamethasone), VIDL (etoposide, ifosfamide, dexamethasone, and L-asparaginase), and DICE (cisplatin, ifosfamide, etoposide, dexamethasone, and L-asparaginase) [3,31-33].

Sandwich chemoradiation combines the concept of sequential chemoradiation followed by additional chemotherapy. This technique has the additional benefit of reducing the intensity of the delivered chemoradiation, which was hypothesized to decrease the toxicity effects seen in other techniques. Wang et al. showed no clinically significant late-onset toxicities in their patients, and their cohort appreciated a two-year survival rate of 86% [12]. Similarly, Li et al. analyzed this technique across three separate Chinese facilities and showed a three-year survival rate of 73%, with only 23% of patients experiencing high-grade leukopenia [13].

It remains unclear which approach, if any, is best as the risk of relapse remains a challenging issue. Recently, Song et al. evaluated the efficacy and toxicity profiles of chemoradiation followed by autologous stem cell transplantation (aSCT) in newly diagnosed advanced ANKL [34]. This study found that VIDL induction with concurrent aSCT was effective with an overall response rate of 74% and mild hematologic toxicity. However, high rates of disease recurrence with secondary relapses in the CNS (67%) have highlighted the need for CNS prophylactic therapies such as methotrexate.

Novel Therapeutic Approaches

Dufva et al. [21] proved that targeting the Janus kinase signal transducer and activator of transcription (JAK-STAT) pathway of malignant NK cells via JAK inhibitors (ruxolitinib) and the BCL-2 inhibitor, venetoclax, allowed for synergistic effects and improved drug sensitivity. Heat shock protein (HSP) 90 inhibitors, polo-like kinase (PLK), cyclin-dependent kinase inhibitors, and histone deacetylase inhibitors were also shown to be effective targets for treatment. 

The polycomb repressive complex 2 (PRC2) receptor is a multiprotein complex that is involved in targeting genes for epigenetic silencing [35]. JAK/STAT pathways may play a role in the overexpression of MYC, which then interacts with PRC2 signaling histone modification [36]. This finding may suggest a novel therapeutic target. 

A current study [37] by New York Medical College and the University of Alabama at Birmingham is evaluating patients with advanced-stage NK lymphoma, NK leukemia, or peripheral T-cell lymphoma in two separate cohorts. Cohort 1 will be treated with a modified SMILE regimen (dexamethasone, methotrexate, ifosfamide, pegaspargase, and etoposide) and pembrolizumab. Cohort 2 will combine pralatrexate (PRX) and brentuximab vedotin (BV) with cyclophosphamide, doxorubicin, and prednisone. Both groups will then undergo allogeneic stem cell transplants. The study is a non-randomized, parallel assignment of approximately 40 patients, which will evaluate the primary outcome of overall response rate with secondary outcomes of progression-free survival.

## Conclusions

Aggressive natural killer-cell leukemia remains a rare, yet devastating disease that does not respond to typical chemoradiation regimens. The continued adaptation of treatment regimens has led to the discovery of multiple techniques that allow for appropriate responses with limited toxicity. Regimens that provide lower dose intensity of chemoradiation with subsequent allogeneic stem cell transplantation have shown some benefits. While progression-free survival rates have had considerable improvement over the past several years, relapse remains an issue. Novel molecular therapeutic targets have shown some promise in vitro by targeting common proteins involved in apoptosis and epigenetic cell silencing. Further clinical trials that utilize these chemoradiation techniques along with transplantation, personalized molecular targeting, and prophylaxis for CNS relapse may achieve the greatest success with these patients.
